# Molecular and biochemical responses of hypoxia exposure in Atlantic croaker collected from hypoxic regions in the northern Gulf of Mexico

**DOI:** 10.1371/journal.pone.0184341

**Published:** 2017-09-08

**Authors:** Md Saydur Rahman, Peter Thomas

**Affiliations:** 1 School of Earth, Environmental and Marine Sciences, University of Texas Rio Grande Valley, Brownsville, Texas, United States of America; 2 Marine Science Institute, University of Texas at Austin, Port Aransas, Texas, United States of America; University of Hawaii System, UNITED STATES

## Abstract

A major impact of global climate change has been the marked increase worldwide in the incidence of coastal hypoxia (dissolved oxygen, DO<2.0 mg l^-1^). However, the extent of hypoxia exposure to motile animals such as fish collected from hypoxic waters as well as their molecular and physiological responses to environmental hypoxia exposure are largely unknown. A suite of potential hypoxia exposure biomarkers was evaluated in Atlantic croaker collected from hypoxic and normoxic regions in the northern Gulf of Mexico (nGOM), and in croaker after laboratory exposure to hypoxia (DO: 1.7 mg l^-1^). Expression of hypoxia-inducible factor-α, *hif-α*; neuronal nitric oxide synthase, *nNOS*; and insulin-like growth factor binding protein, *igfbp* mRNAs and protein carbonyl (PC, an oxidative stress indicator) content were elevated several-fold in brain and liver tissues of croaker collected from nGOM hypoxic sites. All of these molecular and biochemical biomarkers were also upregulated ~3-10-fold in croaker brain and liver tissues within 1–2 days of hypoxia exposure in controlled laboratory experiments. These results suggest that *hif-α*s, *nNOS* and *igfbp-1* transcripts and PC contents are useful biomarkers of environmental hypoxia exposure and some of its physiological effects, making them important components for improved assessments of long-term impacts of environmental hypoxia on fish populations.

## Introduction

Hypoxia (low oxygen <2.0 mg l^-1^) is a major environmental problem in coastal marine ecosystems and a growing global concern [1, 2]. The incidence of coastal hypoxia has increased dramatically worldwide over the past 50 years and now affects over 400 coastal regions covering a total area of ~250,000 km^2^ [1]. However, the long-term impacts on marine ecosystems and fisheries of this increase in coastal hypoxia cannot be accurately assessed due to our current poor understanding of the extent of hypoxia exposure and physiological effects of hypoxia in fish and other motile organisms collected from hypoxic regions.

Hypoxia causes a series of cellular responses including generation of reactive oxygen species (ROS), reactive nitrogen species (RNS), and increased concentrations of nitric oxide synthase (NOS, a RNS regulating enzyme). Hypoxia exposure also causes increased expression of insulin-like growth factor binding protein (IGFBP, a growth inhibitory protein), erythropoietin (EPO), and vascular endothelial growth factor (VEGF) [3–6]. In addition, hypoxia exposure affects the expression of reproductive endocrine genes in teleost fishes such as gonadotropin-releasing hormone-I, luteinizing hormone, and membrane progesterone receptor alpha, resulting in endocrine disruption and marked impairment of reproductive function [3, 4, 7, 8].

Many molecular and physiological responses to hypoxia are regulated by transcription factors named hypoxia-inducible factors (HIFs), heterodimeric proteins with an oxygen-sensitive α-subunit, HIF-α and an oxygen-insensitive β-subunit, HIF-β [3, 5, 6]. HIF-α is a key regulator of many genes such as *igfbp*, *epo*, *vegf* that facilitate adaptation of aquatic organisms to hypoxic environments [5, 6, 9]. Exposure to hypoxia exposure also causes upregulation of HIF-α mRNA expression in tetrapods and teleost fishes [10–12]. Recently, we obtained preliminary evidence that transcript levels of *hif-1*α and *hif-2*α were significantly elevated in ovarian tissues of Atlantic croaker (*Micropogonias undulatus*, a marine fish) collected from hypoxic sites in the northern Gulf of Mexico (nGOM) [3, 8]. Similarly, *hif-1*α and *hif-2*α transcript levels were elevated in liver tissues of dragonet (*Callionymus valenciennei*) collected from hypoxic regions of Tokyo Bay [13]. An important finding was that hypoxia exposure under controlled laboratory conditions caused similar increases in *hif-1*α and *hif-2*α transcript levels in both croaker and dragonet tissues [13, 14]. In addition, *igfbp-1*, *igfbp-2*, neuronal *NOS* (*nNOS*) transcript levels and ROS levels were significantly increased in croaker liver and brain tissues after laboratory exposure to hypoxia [15, 16]. These results suggest that *hif-*α, *igfbp*, *nNOS* and ROS may be useful biomarkers of exposure to environmental hypoxia and its physiological effects in wild teleost populations.

Energy requirements are increased in fish exposed to hypoxia and they adapt by conserving energy expenditure through metabolic suppression [17]. This reduction in the total energy available results in impairment of growth and reproduction which can have long-term ramifications at higher levels of biological organization, leading to a population decline [9, 18]. Consequently, information on the extent of exposure of motile organisms such as fish to environmental hypoxia, and whether they remain in hypoxic bottom waters long enough to elicit physiological adaptive responses, is critical for accurate assessments of the long-term impacts of persistent environmental hypoxia on fish populations. It is not known, for example, whether motile species that feed on benthos such as croaker remain in bottom hypoxic waters or only make brief forays from the overlying normoxic waters to the bottom hypoxic layer for feeding. Therefore, the aim of the present study was to evaluate a suite of potential biomarkers of environmental exposure to hypoxia and its physiological effects in croaker, a relatively hypoxia-tolerant teleost species [3] collected from hypoxic and normoxic bottom water sites in the nGOM, one of the largest seasonal hypoxic regions in the world [1]. Molecular biomarkers of transcriptional responses to hypoxia, *hif-*αs, *hif-β*s, and changes in oxidative status, *nNOS*, were measured in the brain, an oxygen sensitive organ whose functions are highly susceptible to low oxygen levels [6, 16]. A molecular biomarker of growth impairment, *igfbp*, and a biomarker of oxidative stress, protein carbonyl (PC, a ROS [19]), and the hepatosomatic index (HSI, a primary indicator of energy status [20]), were measured in the liver. Nitric oxide levels were also monitored in the plasma. The same suite of biomarkers was measured in controlled laboratory hypoxia experiments to confirm that they are specific responses to hypoxia. The laboratory studies were designed to also provide information on the onset of the response to hypoxia, the magnitude of the response, and its persistence after return to normoxic conditions, information necessary for correct interpretation of the field results. The results show that all these biomarkers were altered in croaker collected from hypoxic regions in the nGOM.

## Materials and methods

### Field sampling and animal collection

**Fig 1:**
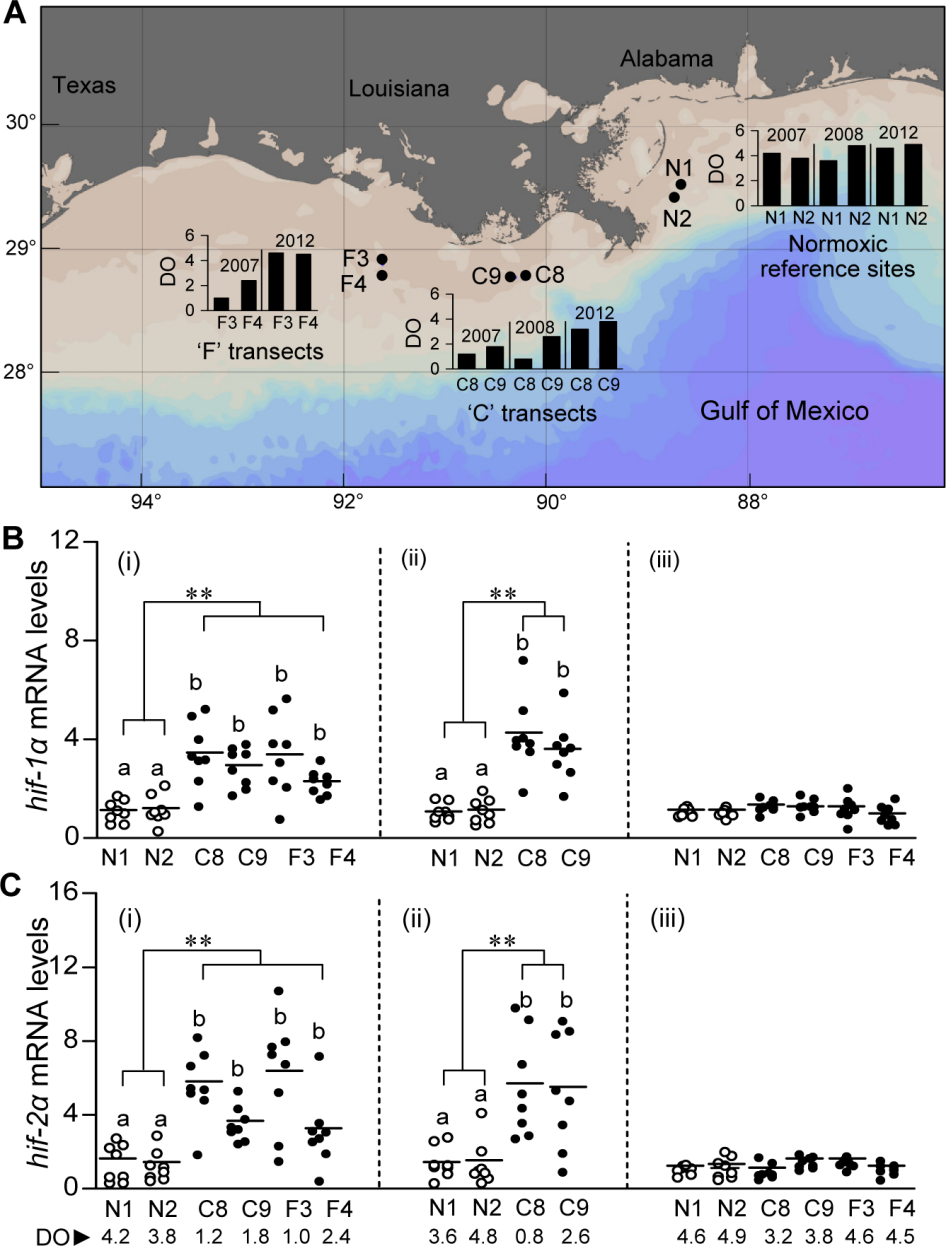
Atlantic croaker sampling sites and brain *hif-α* mRNA levels in croaker collected from hypoxic and normoxic sites in the nGoM. (A) Location of four hypoxic sites (C8, C9, F3 and F4) in the coastal region in the nGoM and two normoxic reference sites (N1 and N2) to the east of the Mississippi Delta where croaker were collected in August, 2007; July, 2008; and August, 2012. The map including sampling sites was generated using Ocean Data View software. Black circles indicate sampling sites. Insert bar graphs indicate dissolve oxygen (DO: mg l^-1^) levels during sampling period. (B, C) Relative *hif-1α* (B) and *hif-2α* (C) mRNA levels in brains of croaker collected from normoxic (N1, N2) and hypoxic (F3, F4, C8, C9) sites in August 2007 (i), July 2008 (ii), and August 2012 (iii) in the nGoM. Asterisks denote significant differences between normoxic (reference) and hypoxic sites (nested ANOVA, **p<0.01). The horizontal lines represent mean values, N = 8. Individual site differences are indicated with different letters (Fisher’s PLSD, *p*<0.05). DO, dissolved oxygen (mg l^-1^).

Immature croaker (~8–9 months old, mean length: 17.4±0.15 cm, body weight, BW: 51.3±1.01 g) were collected over three years in August 2007, July 2008, and August 2012, from either two or four hypoxic sites (C8, C9, F3 and F4; Fig 1A) off the Louisiana coast west of the Mississippi Delta in the northern Gulf of Mexico (nGOM) which are typically hypoxic in the summer months from July to August, and from two sites approximately 180 km east of the Mississippi River Delta which are normally normoxic during the summer [7, 21]. Dissolved oxygen (DO) and other physico-chemical parameters were taken at the time of collection with sensors attached to a conductivity, temperature and depth (CTD) instrument (Fig 1A, S1 Table). Fish were collected using bottom trawls as described previously [7]. Brain and liver tissues were collected from 20 fish at each sampling site, quickly frozen in liquid nitrogen, transported to the University of Texas Marine Science Institute on (UTMSI) dry ice and stored at -80°C or up to 1 month until analyzed.

### Laboratory hypoxia and recovery experiments

Immature croaker were purchased from local fishermen near Port Aransas, Texas in May, and acclimated for two months in recirculating experimental seawater tanks (salinity 30–32‰) at the UTMSI under controlled photoperiod (12L:12D) and temperature (27°C) conditions. Each experimental tank was connected to an external biofilter system. A detailed account of the hypoxia exposure and recovery methods used in this study has been described previously [14, 15]. Briefly, the DO levels in the hypoxia experimental tanks were lowered over a 2-day period by gradually reducing the aeration until the DO levels reached 1.7 mg l^-1^. Fish were continuously exposed to hypoxia (DO: 1.7 mg l^-1^) or normoxia (DO: 6.5 mg l^-1^) for 1 week and fed chopped shrimp daily (3% BW/day). After 1 week of hypoxia exposure, the DO levels in the hypoxia-exposure tanks were re-oxygenated by increasing the aeration which restored DO to normal saturated levels within 2 h, and fish were sampled 3, 6, 12 and 24 hours after re-oxygenation. At the end of the experiments, fish were euthanized under anesthesia using tricaine methanesulfonate (MS-222, Sigma-Aldrich, St. Louis, MO) and blood was collected for measurement of nitrates and nitrites (NOx, a metabolite of NO which reflects the index of total NO production) levels. Brain and liver tissues were rapidly excised, snap frozen in liquid nitrogen, and stored at -80°C for up to 1 month for subsequent measurement of mRNA levels, protein expression, and protein carbonyl (PC) contents.

### Ethics statement

Fish were collected in the nGOM by the research vessel *Pelican* (RV *Pelican*, Louisiana Universities Marine Consortium, LUMCON, Louisiana) which has a permit (permit# SCP34) to collect wildlife species for scientific purposes and abides by the capture rules and regulations issued by Louisiana Department of Wildlife and Fisheries. The field sampling in the nGOM off the Louisiana coastline did not involve threatened or endangered species. All the laboratory experimental procedures were approved by the University of Texas at Austin Institutional Animal Care and Use Committee (IACUC, protocol# 09022701) and followed standard rules and regulations by the IACUC on Animal Care guidelines on the use of animals in the laboratory experiments.

### Quantitative real-time polymerase chain reaction (qRT-PCR) for gene expression

qRT-PCR analyses were performed using an Eppendorf Mastercycler RealPlex (Eppendorf, Hamburg, Germany) on total RNA and croaker hypoxia-inducible factor-αs (*hif-α*), *hif-β*, neuronal nitric oxide synthase (*nNOS*) and insulin-like growth factor binding protein (*igfbp*) gene-specific primers (S2 Table) using a one-step SYBR Green (Agilent Technologies, La Jolla, CA) qRT-PCR method as described previously [15, 16]. Briefly, RNA was extracted from liver and brain tissues using TRI reagent (Sigma-Aldrich), treated with DNase (Promega, Madison, WI), and quantified with a NanoDrop (Thermo Fisher Scientific, Waltham, MA). qRT-PCR analyses were performed in a 25 μl reaction mixture containing 12.5 μl of 2x SYBR Green master mix, 1 μl of reverse transcriptase enzyme, 100 ng of RNA, and 125 nM of forward and reverse primers. Each transcript level was normalized of the quantification of croaker *18S* rRNA (primers: forward 5’-AGAAACGGCTACCACATCCA-3’ and reverse 5’-TCCCGAGATCCAACTACGAG -3’; GenBank accession number: AY866435). The relative mRNA levels were analyzed using 2^-ΔΔC*t*^ method [22].

### Western blot analysis for nNOS protein expression

Protein was extracted from hypothalamic tissues with a TRI reagent-guanidine technique according to Rodrigo et al. [23], an optimized methodology for sequential extraction of RNA and protein from a single sample [16]. Briefly, protein samples (2 μg) were solubilized by boiling in loading buffer and cooled on ice for 5 min. Solubilized proteins were electrophoresed on 10% sodium dodecyl sulfate (SDS) polyacrylamide gels, transferred onto polyvinylidene difluoride (PVDF) membranes, blocked with 5% nonfat dry milk, and incubated with a primary nNOS antibody (dilution: 1:1,000) overnight at 4°C. The *nNOS* gene and antibody have been fully characterized and validated in croaker brain previously [16]. PVDF membranes were then incubated with secondary antibody (Southern Biotech, Birmingham, AL), visualized by an enhanced chemiluminescence substrate (Pierce, Rockford, IL) and exposed to X-ray film (Amersham Biosciences, Buckinghamshire, UK). The intensity of the protein bands was estimated using ImageJ software (NIH, Bethesda, MD). Actin protein was also used as an internal control to normalize sample loading on the gels.

### Measurement of plasma NOx concentration

Quantification of NO is problematic due to its short life time [24]. Therefore, the final and stable end products of NO in nitrates and nitrites (NOx) were measured with a colorimetric assay using Griess reagent according to the manufacturer’s protocols (Enzo Life Sciences, Plymouth Meeting, PA). Briefly, plasma samples (150 μl) were deproteinized by adding 150 μl of trichloroacetic acid (10% solution) (Sigma Aldrich), vortexed for 30 s and placed in a dark chamber for 15 min. The samples were then centrifuged at 14,000×*g* for 5 min and the supernatant was removed for analysis. The assay procedure consisted of adding 50 μl of standard or sample to a 96-well microplate containing 50 μl of reaction buffer, 25 μl of diluted nicotinamide adenine dinucleotide hydrogen (NADH) and 25 μl of nitrate reductase. The plate was sealed and incubated for 30 min at 37°C in the dark with gentle shaking. Fifty μl of Griess reagent-I and -II were added into each well and the plate was incubated at room temperature for 10 min in the dark. The optical density (OD) was assayed using a microplate reader (Fluoro Star Optima, BMG Labtechnologies, Durham, NC) and expressed as nmol ml^-1^.

### Measurement of protein carbonyl (PC) contents

PC contents were measured in liver homogenate supernatants by reacting samples with 2,4-dinitrophenylhydrazine to generate dinitrophenyl-hydrazones according to the method of Reznick and Packer [25]. Briefly, 200 μl supernatant was added to each of two 2 ml microcentrifuge tubes containing 800 μl of 2.5 M hydrochloric (HCl) acid in the presence or absence of 800 μl of dinitrophenylhydrazine (DNPH) solution. The sample mixture was then incubated in the dark at room temperature for 1 h. After the incubation, the proteins were precipitated with the addition of 1 ml 20% trichloroacetic acid (TCA) and centrifuged at 10,000x *g* for 10 min at 4°C. The resulting pellet was washed three times with 1 ml of ethanol:ethyl acetate solution (1:1) and centrifuged between washes to remove excess DNPH. After a final wash, the protein pellet was solubilized in 500 μl of 6 M guanidine hydrochloride (Sigma-Aldrich). The PC contents were determined from the difference between the average absorbance (λ = 370 nm) of duplicate samples treated with DNPH and not treated with DNPH using a Fluoro Star spectrophotometer (BMG Labtechnologies). The PC contents were expressed as nmol mg^-1^ protein.

### Statistical analyses

Significant differences between field data for normoxic and hypoxic sites were analyzed by nested analysis of variance (ANOVA), and laboratory data by one-way ANOVA. Where significant interactions were found, this was followed by Fisher’s protected least-significant difference (PLSD) test for multiple comparisons and Student’s *t*-test for unpaired comparisons. A *p* value <0.05 was considered statistically significant for all the tests. Analyses were performed using SYSTAT (Systat, San Jose, CA), Statview (SAS Institute Inc., Cary, NC) and GraphPad Prism (GraphPad, San Diego, CA) computer software.

## Results

### Hypoxic status at the sampling sites

In the earlier field samples collected from C and F transects in the northern nGOM hypoxic zone from 2007 to 2011, we and other researchers found that the C8 and C9 transects and the F3 and F4 transects remained hypoxic (mean DO: 1.6 mg l^-1^ in C and 1.7 mg l^-1^ in F transects) throughout the summer months until mid-September (S3 and S4 Tables) (http://accession.nodc.noaa.gov/0117436, files in ISO 19115–2 Metadata [7, 8, 26–28]). In 2012, however, the size of the hypoxic zone in the nGOM was greatly reduced due to a reduction in Mississippi river water flow associated with drought in mid-western regions in the United States (http://www.noaanews.noaa.gov/stories2012/20120727_midwestdrought.html [29]). The DO levels in the bottom water at C and F transects were significantly higher during our sampling times in 2012 (mean DO: 3.5 mg l^-1^ in C and 4.6 mg l^-1^ in F transects) than in 2007 (mean DO: 1.5 mg l^-1^ in C and 1.7 mg l^-1^ in F transects) and 2008 (mean DO: 1.7 mg l^-1^ in C transect) (Fig 1A). Similarly, other researchers also found that the C8, C9 and F4 sites and neighboring transects remained normoxic in 2012 (mean DO: 4.13 mg l^-1^, S5 and S6 Tables). Therefore, the data on hypoxia biomarkers in croaker collected from these sites in 2012 would provide a useful comparison with those collected in 2007 and 2008 when the DO levels were much lower.

### Hypoxia-induced HIFs regulation in croaker brain: field and laboratory findings

The mean *hif-1α* mRNA levels in croaker brains were significantly higher (~2.5-3-fold) in fish collected from the hypoxic sites, C8, C9, (mean DO: 1.6 mg l^-1^) compared to the reference normoxic sites, N1 and N2 (mean DO: 4.1 mg l^-1^) in 2007 and 2008 (Fig 1B-i and 1B-ii) and in croaker collected in 2007 from hypoxic sites F3 and F4 compared to controls (Fig 1B-i). In contrast, the mean *hif-1α* mRNA levels were not significantly elevated at the C and F sites in 2012, when DO levels were higher (mean DO: 3.5–4.6 mg l^-1^) (Fig 1C-iii). Similarly, there was a significant ~4-6-fold increase in *hif-2α* mRNA levels in croaker brains collected from hypoxic sites compared with brain levels in fish collected from normoxic reference sites in 2007 and 2008 (Fig 1C-i and 1C-ii), but brain *hif-2α* mRNA levels were not significantly different in fish collected in 2012 from the C and F sites than those at the reference sites (Fig 1C-iii). Transcript levels of *hif-1α* and *hif-2α* in brains of croaker collected from all three sites in 2012 were uniformly low (1.12±0.06) and similar to those in croaker at the normoxic sites in 2007 and 2012. In contrast, expression of *hif-1α* and *hif-2α* mRNAs was more variable at the hypoxic sites in 2007 and 2008 (4.06±0.22) and several individuals had low mRNA levels similar to those at the normoxic sites. There was no significant elevation of *hif-1β* and *hif-2β* mRNA levels in croaker brains from hypoxic sites compared with those from reference normoxic sites in 2007 and 2008, and they were similar at all the sites in 2012 (S1A and S1B Fig).

**Fig 2:**
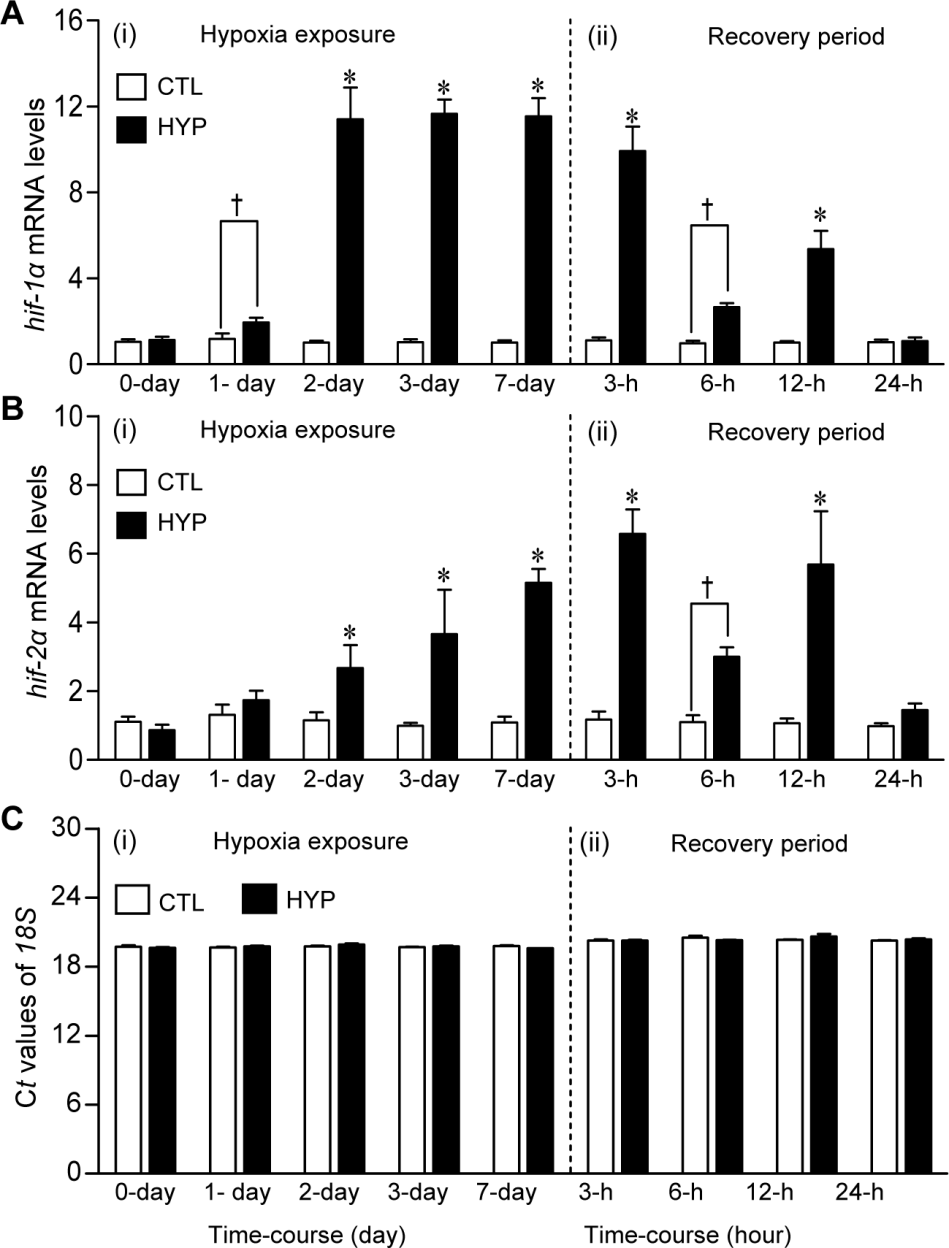
Expression of *hif-1α* and *hif-2α* mRNA levels in brains of Atlantic croaker exposed to laboratory hypoxia. Effects of 7-day laboratory exposure to normoxia control (CTL, dissolved oxygen, DO: ~6.5 mg l^-1^, white bars), hypoxia (HYP, DO: 1.7 mg l^-1^, black bars) and a recovery period on relative *hif-1α* (A-ii) and *hif-2α* (B-ii) mRNA levels, and (C) C*t* values of *18S* rRNA in croaker brains. Note: here and in subsequent figures laboratory exposure duration only refers to period fish were exposed to target DO; fish were previously exposed to declining DO for additional 2-day adjustment period. After 7-day hypoxia exposure, DO of hypoxic treatment was restored to normoxic level (24-h recovery period). Each bar represents the mean±SEM (N = 7–8). Differences in the relative mRNA levels between the start of the experiment (CTL, control) and each treatment were tested by Dunnett’s test, *p<0.05. ‘†’ indicates significant difference from normoxic controls (Student’s *t*-test, *p*<0.05). C*t*, threshold cycle.

The time-course of changes in *hif-α*s and *hif-β*s transcript levels was investigated under both normoxic and hypoxic conditions in controlled laboratory experiments (Fig 2A and 2B and S2A and S2B Fig). There were no significant changes in the relative mRNA levels of both *hif-1α* and *hif-2α* in croaker brains in the normoxic control group during the experimental period (Fig 2A and 2B). The transcript levels of *hif-1α* increased significantly (~1.5-fold) after 1 day exposure to hypoxia (DO: 1.7 mg l^-1^), were maximal (~10-fold control levels) after 2 days of hypoxia exposure, and remained elevated in all individuals for up to 7 days of continuous hypoxia exposure (Fig 2A-i). The transcript levels of *hif-2α* did not change significantly until the second day of hypoxia exposure and increased gradually until they reached ~5-fold control values after 7 days of hypoxia exposure (Fig 2B-i). The *hif-1α* and *-2α* mRNA levels declined rapidly in the hypoxia-exposed fish after the DO levels in the tanks were restored to normal, saturated levels and were not significantly different from controls 24 h after the cessation of hypoxia exposure (Fig 2A-ii and 2B-ii). Similar to the results of the field studies, *hif-1β* and *hif-2β* mRNA levels in croaker brains were unaltered in the laboratory experiments after hypoxia exposure (S4A and S4B Fig).

### Hypoxia-induced nNOS regulation in croaker brain: Field and laboratory findings

**Fig 3:**
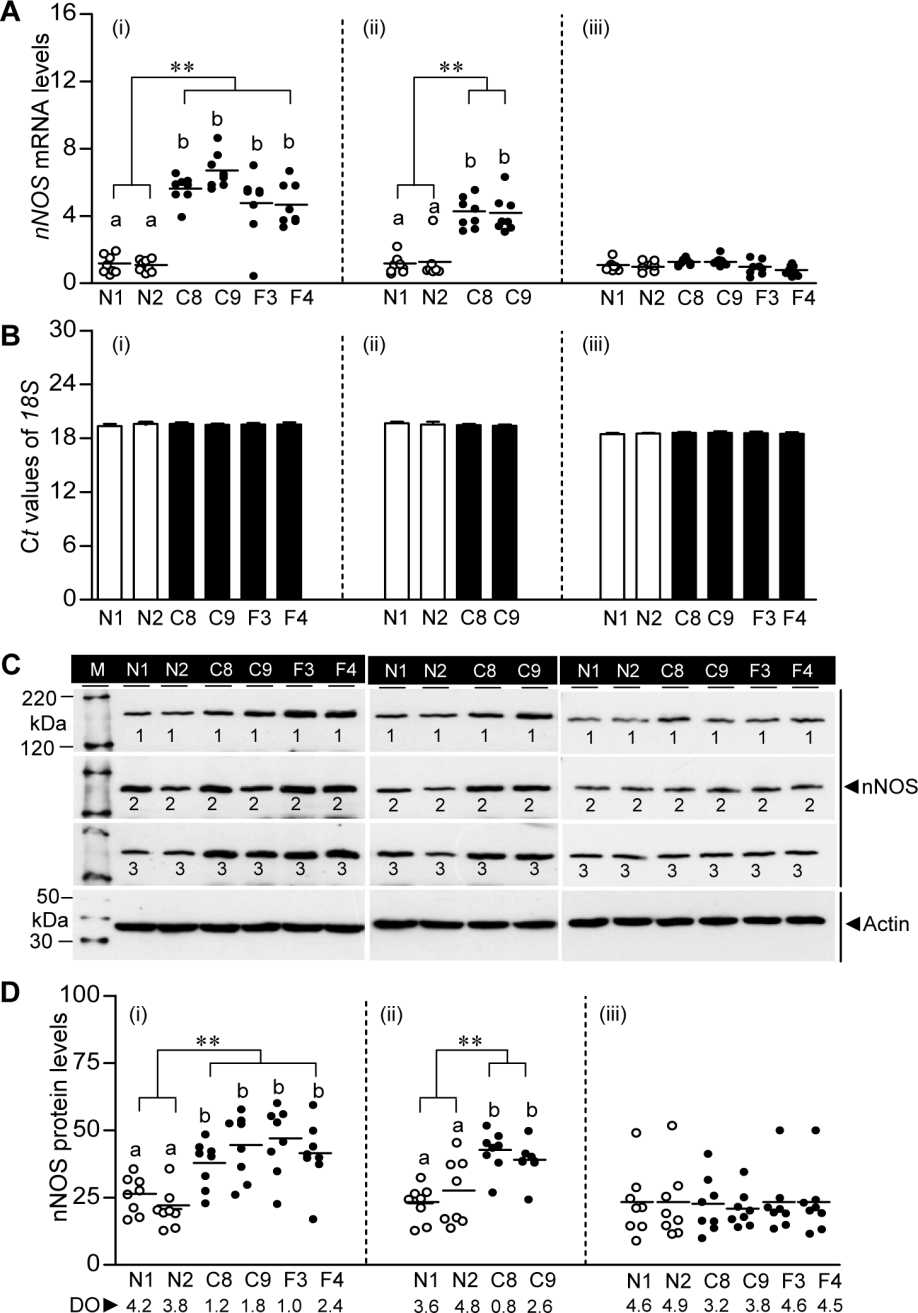
Brain *nNOS* mRNA and protein levels in Atlantic croaker collected from hypoxic and normoxic sites in the nGoM. Relative *nNOS* mRNA levels (A), (B) C*t* values of *18S* rRNA, and nNOS protein expression and levels (C,D) in brains of croaker collected from normoxic (N1, N2) and hypoxic (F3, F4, C8, C9) sites in August 2007 (i), July 2008 (ii), and August 2012 (iii). Representative Western blot of nNOS protein expression in croaker brain samples (B). The horizontal lines represent mean values, N = 8. A nested ANOVA indicates *nNOS* mRNA and protein levels in croaker from the normoxic sites were significantly different from those in fish from the hypoxic sites (**p<0.01). Individual site differences identified with a multiple range test, Fisher's PLSD, are indicated with different letters. M, marker; kDa, kilodalton.

*nNOS* mRNA levels were significantly increased in the brains of croaker collected from the hypoxic sites compared to those in fish collected from the normoxic sites (N transect) in 2007 and 2008 (Fig 3A-i and 3A-ii). Transcript levels of *nNOS* in croaker collected from the hypoxic sites were less variable than those of *hif-1α* and *hif-2α* and were uniformly higher than those of fish from the normoxic sites. Similarly, nNOS protein levels were elevated in brains of croaker collected from hypoxic sites in 2007 and 2008 compared to the concentrations in brains of croaker collected from the normoxic sites (Fig 3B-i and 3B-ii). However, there was no significant difference in *nNOS* mRNA and protein values in brains of croaker collected from any of the sites in 2012, which were similar to those from normoxic sites in 2007 and 2008 (Fig 3A-iii and 3C-iii).

**Fig 4:**
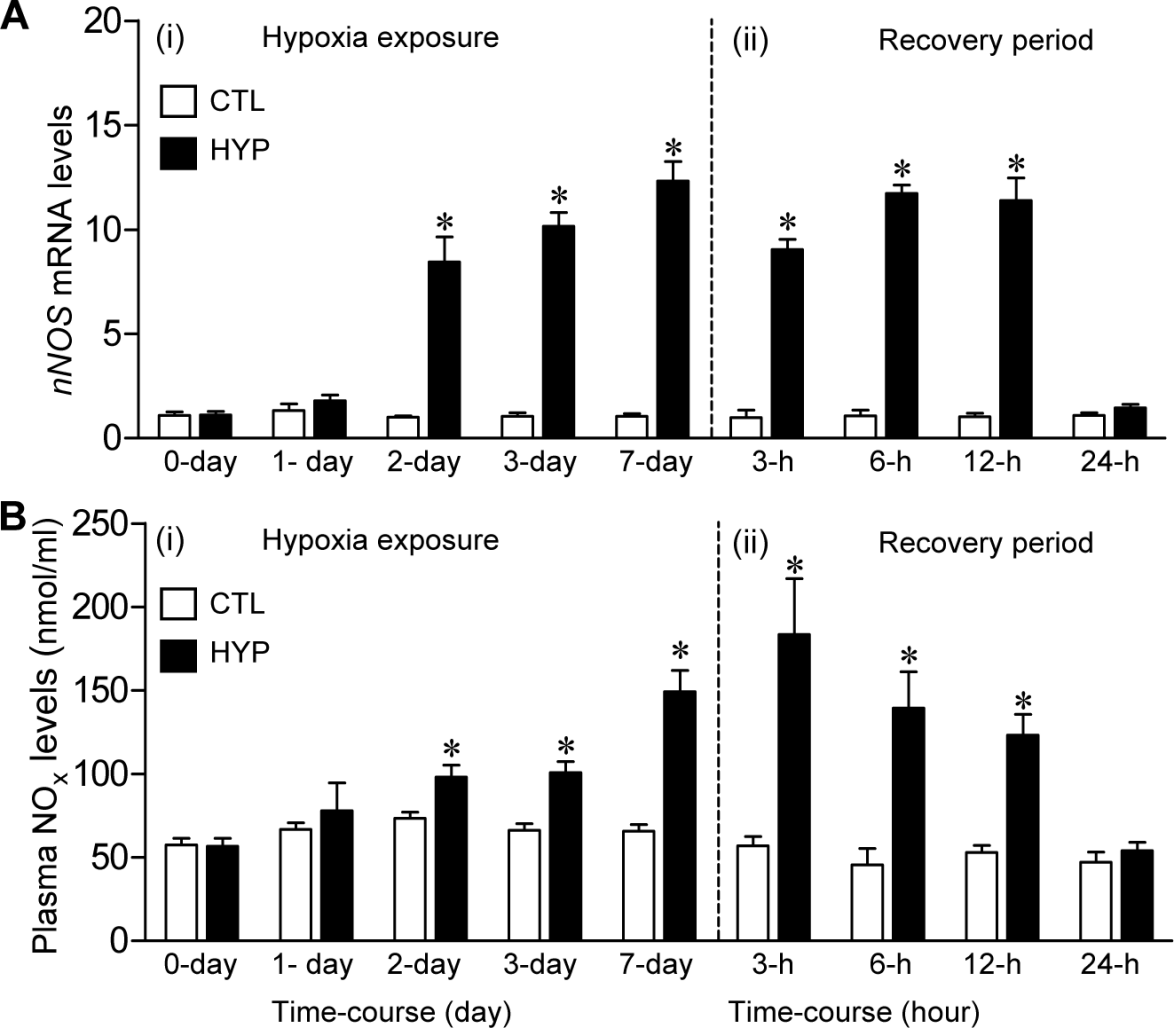
Expression of *nNOS* mRNA in the brains and plasma NOx levels in Atlantic croaker exposed to laboratory hypoxia. Effects of 7-day laboratory exposure to normoxia (dissolved oxygen, DO: ~6.5 mg/L, white bars), hypoxia (HYP, DO: 1.7 mg l^-1^, black bars) and a recovery period on hypothalamic *nNOS* mRNA levels (A) and plasma NO metabolites, nitrite plus nitrate (NOx) levels (B). Each value represents the mean±SEM (N = 7–11). Differences in the relative mRNA levels between the start of the experiment (CTL, control) and each treatment were tested by Dunnett’s test, *p<0.05.

*nNOS* mRNA and plasma nitric oxide metabolite (NOx) levels were significantly elevated 8-fold and 1.2-fold, respectively, after 2 days of hypoxia exposure in controlled laboratory conditions (Fig 4A-i and 4B-i) and declined to control levels within 24 h of restoration to normoxic conditions (Fig 4A-ii and 4B-ii).

### Hypoxia-induced IGFBP regulation in croaker liver: Field and laboratory findings

**Fig 5:**
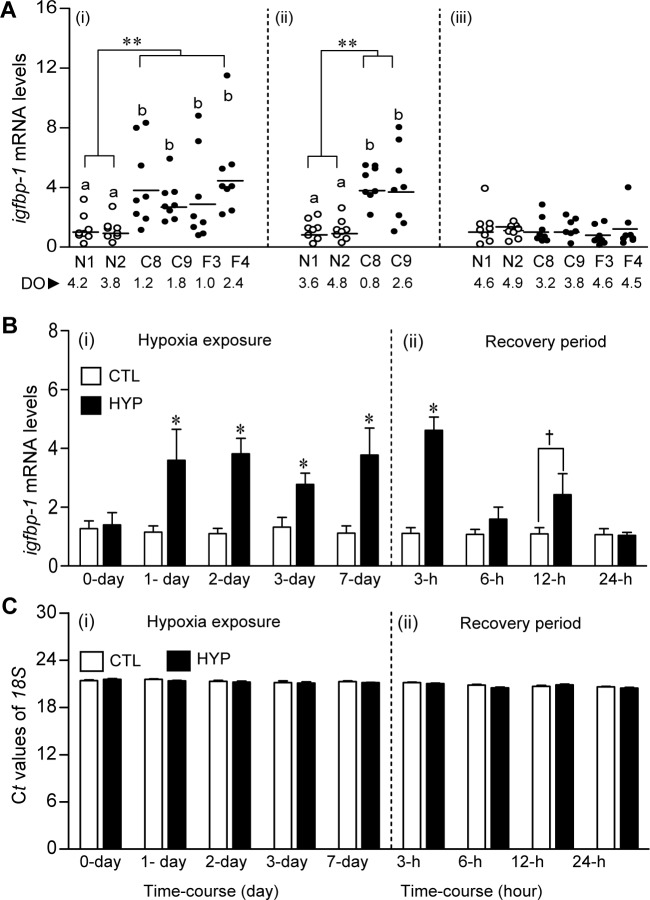
*igfbp-1* mRNA levels in livers of Atlantic croaker collected from normoxic and hypoxic sites in the nGOM and after exposure to hypoxia in the laboratory. Croaker collected from normoxic (N1, N2) and hypoxic (F3, F4, C8, C9) sites in August 2007 (i), July 2008 (ii), and August 2012 (iii) in the nGoM (A). Asterisks denote significant differences between normoxic (reference) and hypoxic sites (nested ANOVA, **p<0.01). The horizontal lines represent mean values, N = 7–8. Individual site differences are indicated with different letters (Fisher’s PLSD, *p*<0.05). (B, C) Expression of *igfbp-1* mRNA levels and C*t* values of *18S* rRNA in livers of croaker exposed to laboratory hypoxia. Effects of 7-day laboratory exposure to normoxia (DO: ~6.5 mg l^-1^, white bars), hypoxia (HYP, DO: 1.7 mg l^-1^, black bars) and recovery period on *igfbp-1* mRNA levels in croaker liver. Each value represents the mean±SE (N = 7–8). Differences in the relative mRNA levels between the start of the experiment (CTL, control) and each treatment were tested by Dunnett’s test, *p<0.05. ‘†’ indicates significant difference from normoxic controls (Student’s *t*-test, *p*<0.05).

To assess whether exposure to environmental hypoxia alters a biomarker of growth impairment, *igfbp-1* and *igfbp-2* mRNAs were assayed in croaker liver. *igfbp-1* mRNA levels were significantly higher in the liver tissues of croaker collected from the hypoxic sites compared to those in fish from the normoxic sites in 2007 and 2008, but were low and not significantly different at any of the sites in 2012 when DO levels were higher (Fig 5A-i and 5A-ii). In contrast, there were no significant changes of *igfbp-2* mRNA levels in croaker liver tissues collected from the hypoxic and normoxic sites in 2007 and 2008, or at any of the sites in 2012 (S3A Fig).

The time-course of changes in *igfbp* mRNA expression in croaker livers was investigated in a laboratory hypoxia exposure study. *igfbp-1* mRNA levels significantly increased (~3.9-fold) in croaker livers after 1 day of hypoxia exposure in controlled laboratory conditions and decreased to control levels within 24 h of restoration to normoxic conditions (Fig 5B). There were no significant changes of *igfbp-2* mRNA levels in liver tissues compared to controls after 1- to 7 days of hypoxia-exposure (S5B Fig).

The hepatosomatic index (HSI: [liver weight/BW] x 100) is most commonly used to provide an indication of the energy store (fat, lipid and protein) in animals [20] which is generally reduced under hypoxic conditions [30]. HSI was significantly lower in livers of croaker collected from hypoxia sites compared to those in fish collected from normoxic sites in 2007 and 2008, whereas they were similar in 2012 at all the field sites, indicating low hepatic energy reserves in croaker at the hypoxic sites in 2007 and 2008 (S4-I and S4-ii Fig).

### Hierarchical clustering of relative gene expressions in croaker brain and liver: Field and laboratory findings

**Fig 6:**
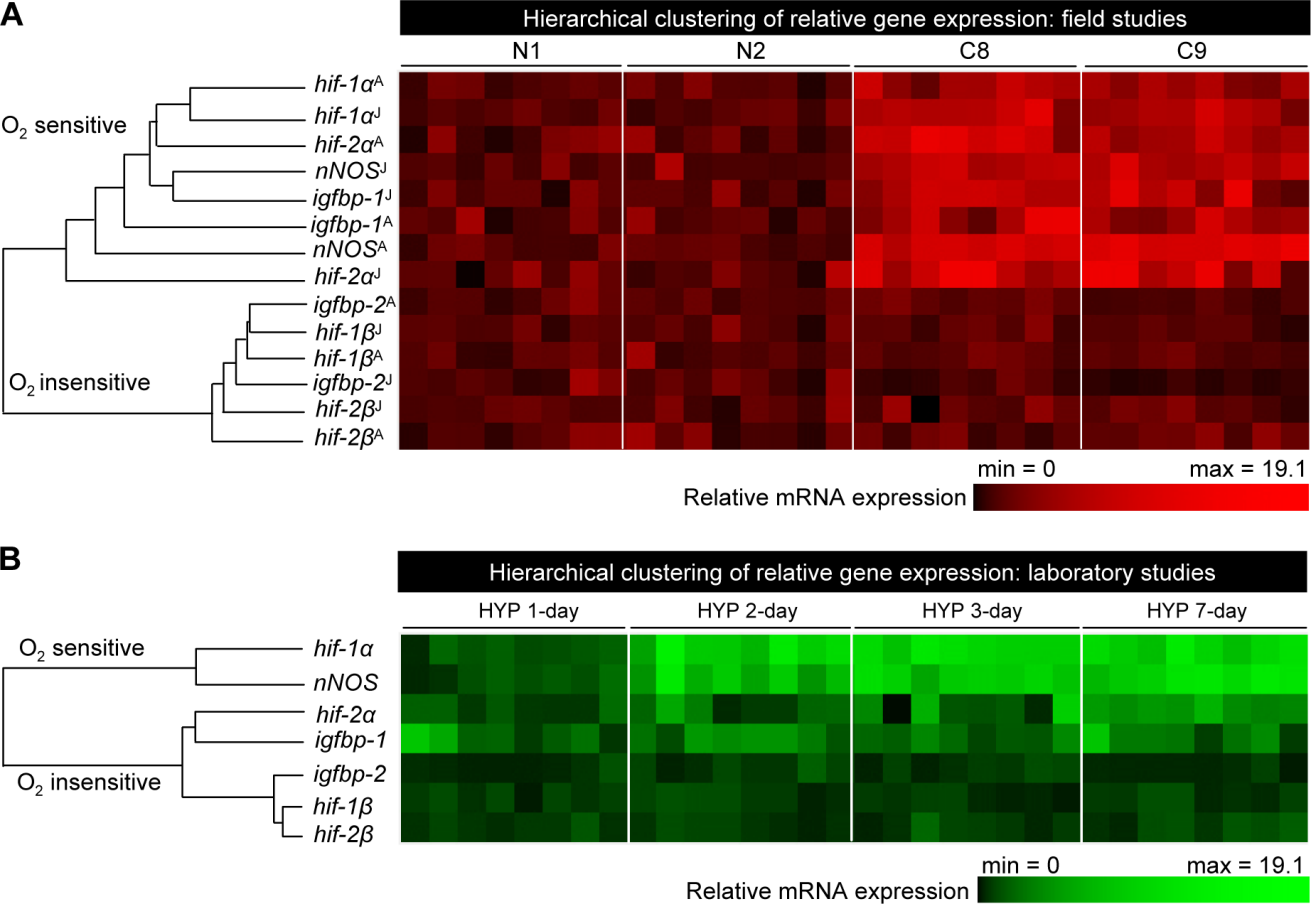
Hierarchical clustering of gene expression in the brain and liver tissues of Atlantic croaker exposed to environmental and laboratory hypoxia. Croaker collected from normoxic (N1, N2) and hypoxic (C8, C9) sites in August 2007 and July 2008 in the nGoM (A) and laboratory hypoxia experiment (B). ^A^: August; ^J^: July.

Gene expression clustering is one of the most useful techniques to organize and cluster genes according to similarities in their expression profiles [31]. Hierarchical clustering of relative gene expression of croaker *hif-α*s, *hif-β*s, *nNOS* and *igfbp*s showed different variations among the different oxygen regimens in fish collected from hypoxic and normoxic sites in the nGOM in 2007 and 2008 (Fig 6A). Two clades were found; the first clade, the oxygen-sensitive gene group, showed a close relationship and co-variation in the expression of *hif-α*s, *nNOS* and *igfbp-1*, where *hif-α*s distinctly displayed more similarity with *nNOS* than *igfbp-1*. The second clade, the oxygen-insensitive group, clustered *hif-β*s and *igfbp-2* (Fig 6A).

During the time-course laboratory hypoxia experiments, two clades were also found (Fig 6B). The first clade, the oxygen-sensitive group, showed two subgroups; the first subgroup clustered *hif-1α* and *nNOS* while the second subgroup clustered *hif-2α* and *igfbp-1*. The second clade, the oxygen-insensitive group, clustered *hif-β*s and *igfbp-2*.

### Hypoxia-induced protein carbonyl (PC) contents in croaker liver: Field and laboratory findings

**Fig 7:**
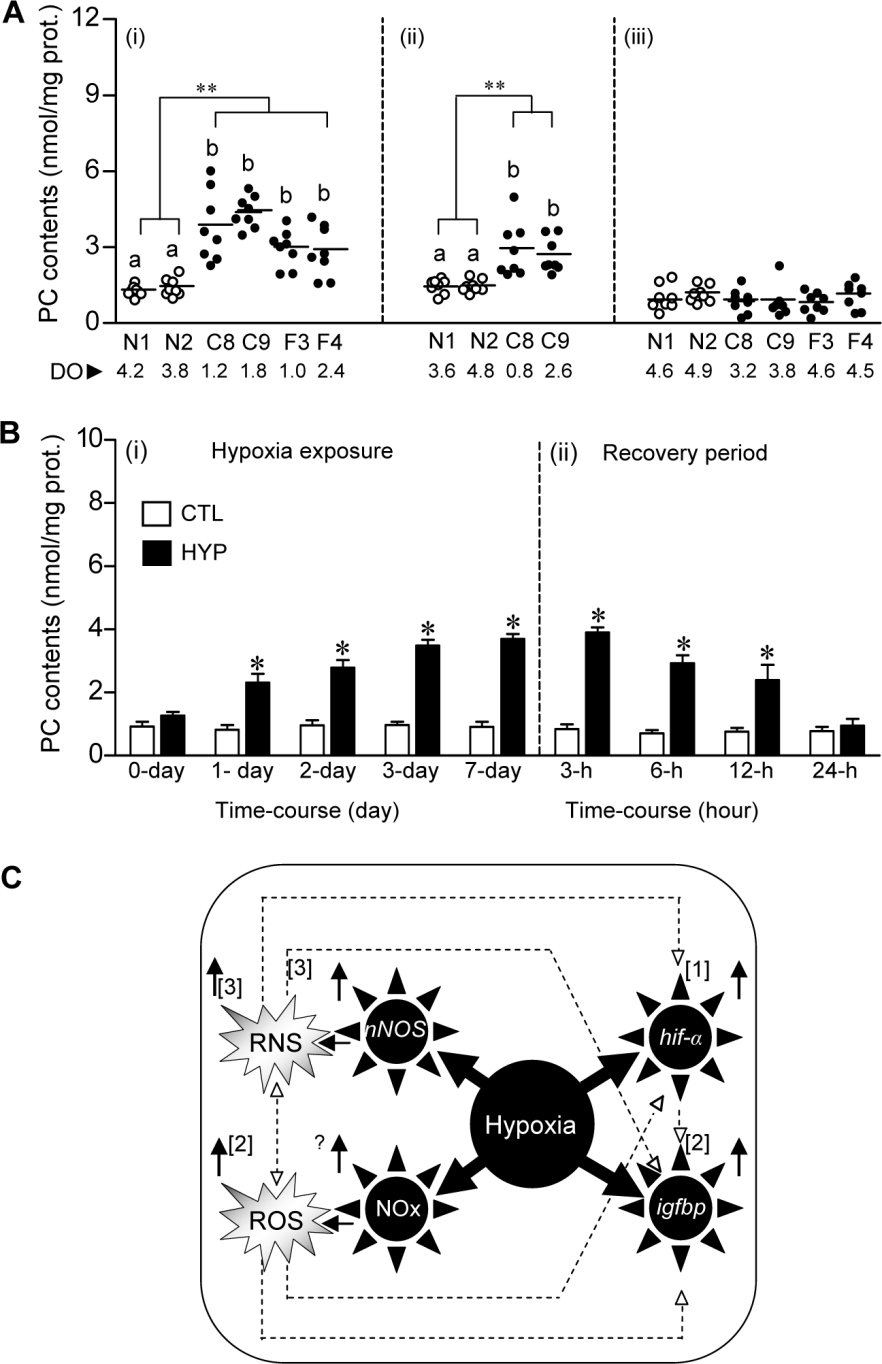
Protein carbonyl (PC) contents in Atlantic croaker exposed to environmental and laboratory hypoxia. (A) PC contents in croaker liver collected from normoxic (N1, N2) and hypoxic (F3, F4, C8, C9) sites in August 2007 (i), July 2008 (ii) and August 2012 (iii) in the nGoM. The horizontal lines represent mean values, N = 8. A nested ANOVA indicates PC contents in croaker liver from the normoxic sites were significantly different from those in fish from the hypoxic sites (**p<0.01). Individual site differences identified with a multiple range test, Fisher's PLSD, are indicated with different letters. DO, dissolved oxygen (mg l^-1^). (B) Effects of 7-day laboratory exposure to normoxia (DO: ~6.5 mg l^-1^, white bars), hypoxia (HYP, DO: 1.7 mg l^-1^, black bars) and recovery period on PC contents in croaker liver. Each value represents the mean±SE (N = 7–8). Differences in the PC contents between the start of the experiment (CTL, control) and each treatment were tested by Dunnett’s test, *p<0.05. prot., protein. (C) Proposed model of hypoxia-induced upregulation of croaker *hif-α*, *nNOS*, *igfbp* transcripts, NOx, and ROS and RNS (solid arrows pointing up), based on our present and previous studies (^1,2,3^Rahman and Thomas, 2007, 2011, 2013). The model shows several pathways (dotted lines) through which hypoxia could potentially upregulate gene and biochemical biomarkers in croaker. ‘?’: evidence has only been obtained in mammalian *in vitro* studies (Li and Jackson, 2002).

PC contents were significantly higher in the liver tissues of croaker collected from the hypoxic sites compared to those in fish from the normoxic sites in 2007 and 2008, but did not show any differences in fish collected from the three sites in 2012 (Fig 7A). In controlled laboratory time-course hypoxia experiments, PC contents were upregulated ~2-fold after 1 day of hypoxia exposure and declined to control levels within 24 h of restoration to normoxic conditions (Fig 7B).

## Discussion

The results of the present study provide several lines of evidence from both field and controlled laboratory studies that *hif-1α*, *hif-2α*, *nNOS* mRNAs and nNOS protein levels in the brain, and *igfbp-1* mRNA levels and PC contents in the liver tissues of Atlantic croaker are accurate molecular and biochemical biomarkers of environmental hypoxia exposure. The finding that the mean values of all of these biomarkers during two summer seasons were elevated several-fold in croaker collected from hypoxic sites in the nGOM compared to those fish collected from normoxic sites suggests that environmental exposure to hypoxia and its physiological effects can potentially be detected in croaker populations by measurement of this suite of biomarkers. The observation that these site differences in biomarker responses disappeared during a third year when all the sites were normoxic suggests these biomarker responses are hypoxia-specific and not site-specific. Strong evidence that these biomarkers are specifically upregulated by hypoxia exposure was obtained in controlled laboratory studies which showed similar increases in *hif-α*s, *nNOS* and *igfbp-1* mRNA levels and PC contents in croaker brain and liver tissues after constant laboratory exposure to the same DO levels as those measured at the hypoxic field sites. The fact that genes closely related to these biomarkers, *hif-1β*, *hif-2β*, and *igfbp-2*, were not increased in croaker tissues collected from the hypoxic sites in the nGOM or after laboratory hypoxia exposure indicates that only a subset of genes are specifically upregulated by hypoxia. The similar expression profiles of the hypoxia biomarkers in croaker, that is the oxygen-sensitive genes *hif-α*s, *nNOS*, *igfbp-1*, in the field and laboratory studies was confirmed by hierarchical clustering. The oxygen-insensitive genes, *hif-β*s and *igfbp-2*, also showed similar profiles in the laboratory and field studies as revealed by hierarchical cluster analysis. The laboratory results also show the time-course of the biomarkers responses to hypoxia exposure, information that is critical for interpreting the field data and for providing an estimate of the duration of hypoxia exposure of croaker at the hypoxic sites. Taken together, these results provide the necessary background and validation of *hif-1α*, *hif-2α*, *nNOS* and *igfbp-1* mRNAs and nNOS protein levels, and PC contents in croaker tissues for their use as specific and reliable biomarkers of environmental exposure to hypoxia.

The finding that the transcript levels of *hif-1α* and *hif-2α* in the brains of croaker collected over two years from hypoxic sites in the nGOM were significantly elevated compared to those from normoxic sites is consistent with preliminary evidence obtained in previous studies that these genes were upregulated in ovaries of croaker collected from hypoxic estuarine and coastal regions of the northern GOM [3, 7]. Similarly, tissue levels of *hif-1α* and *hif-2α* mRNAs were increased in dragonet [13], and in mantis shrimp [32], collected from hypoxic areas in Tokyo Bay. Collectively, these field studies provide strong evidence that the upregulation of *hif-α*s transcripts is a widespread response to environmental hypoxia in marine organisms, and consequently is of broad utility as a molecular biomarker of hypoxia exposure.

The results from the controlled laboratory studies on the time-courses of the biomarker responses to hypoxia exposure provide valuable insights into the likely recent hypoxia exposure histories of croaker collected in the nGOM. For example, the observation that *hif-1α* and *hif-2α* mRNA levels were increased several-fold in croaker brains after 2 days of laboratory hypoxia exposure suggests that the croaker collected from the nGOM with substantially elevated *hif-α*s mRNA levels had likely been exposed to hypoxic conditions for at least 2 days. Moreover, it is highly likely that these fish had recently been exposed to hypoxic conditions because we found that croaker *hif-1α* and *hif-2α* mRNA levels declined to control levels within 24 hours of restoration to normoxic conditions in the laboratory study. Similar upregulation of *hif-1α* and *hif-2α* mRNA expression after 7 days of hypoxia exposure and a rapid return to pre-exposure levels within 24 hours of restoring normoxic conditions in the laboratory study have been reported in dragonet and mantis shrimp [13, 32]. In addition, upregulation of *hif-1α* mRNA expression after 8 hours of hypoxia exposure and return to baseline levels within 24 hours have also been reported in another marine fish, Pacific herring (*Clupea pallasii*) [33]. Together these laboratory results suggest that there is a common response pattern of *hif-α*s transcripts in marine organisms to hypoxia exposure.

Interestingly, *nNOS* mRNA and plasma NO displayed the same time-course of changes in expression as *hif-α* mRNA levels after laboratory hypoxia exposure in croaker, whereas hepatic *igfbp-1* transcript levels and PC contents increased more rapidly and were markedly elevated within 1 day of hypoxia exposure and returned to baseline values within 6 hours and 24 hours, respectively, of return to normoxic conditions. These differing patterns of biomarker expression have the potential to further delineate the hypoxia exposure histories of croaker collected from hypoxic sites. For example, upregulation of *igfbp-1* mRNA and PC content in an individual in the absence of changes in *hif-α* and *nNOS* mRNA expression might indicate its recent arrival 1 day earlier in hypoxic waters. An initial comparison of the individual *hif-1α* mRNA levels shows several samples collected from the hypoxic field sites had low *hif-1α* expression similar to that at the normoxic sites (Fig 1B), whereas the *hif-1α* mRNA levels were consistently elevated in individuals exposed to hypoxia in the laboratory (S5 Fig). The results suggest that upregulation of *hif-1α* is an ubiquitous response in croaker to hypoxia exposure and that individuals at the hypoxic sites with low *hif-1α* levels had only recently arrived there from a normoxic area. Thus, measurement of certain biomarkers may also provide estimates of the percentage of fish collected from hypoxic sites that had recently been exposed to hypoxia which is important for assessing the impacts of environmental hypoxia on fish populations. It cannot be determined from the present results whether croaker at the hypoxic sites in the nGOM were continuously exposed to hypoxia, or migrated periodically out of the hypoxic bottom waters into the surrounding normoxic water higher in the water column. Nevertheless, the fact remains that their hypoxia exposure in croaker collected from these sites is sufficient to significantly increase their *hif-1α* and *hif-2α* expression, which results in profound physiological and metabolic changes that potentially have long-term population impacts.

Exposure to hypoxia increases nNOS activity in vertebrate brains resulting in over production of NO which leads to oxidative damage and neuronal dysfunction [34]. It is likely that this increased NO generation observed in the present study as shown plasma increased levels of NOx contributes to the marked impairment of reproductive neuroendocrine functions in croaker observed under hypoxic conditions, because both eNOS activity and NO production are reversed by co-treatment with antioxidants [16, 35]. The current study suggests a similar relationship between increased nNOS activity and neuroendocrine impairment may occur in croaker exposed to environmental hypoxia in the nGOM because *nNOS* mRNA and protein levels were significantly higher in the brains of croaker collected from hypoxic sites compared to fish from the normoxic sites, the same sites where marked impairment of neuroendocrine reproduction functions was observed in croaker collected a few months later in 2007 [7]. Upregulation of brain *nNOS* mRNA expression is likely a common response of teleost fish to hypoxia exposure because a similar increase in *nNOS* mRNA levels was reported in the brains of rainbow trout (*Oncorhynchus mykiss)* after 4 days of exposure to reduced DO conditions which was accompanied by increases in plasma NOx levels [24].

The field study results corroborate our present and previous laboratory findings with croaker [15] that *igfbp-1* transcript levels are consistently upregulated by hypoxia exposure, while *igfbp-2* mRNA levels are unchanged. Collectively, these results suggest that *igfbp-1* is the major *igfbp* gene involved in early adaptation of fishes to hypoxia stress. Indeed, IGFBP-1 is highly inducible under catabolic and stress conditions such as starvation, injury and hypoxia [36–38]. Recent studies have indicated that *igfbp-1* serves as a molecular switch in fishes by impairing IGF signaling and redirecting energy resources from growth towards metabolic processes critical for survival such as glycolysis [39].

The results show that oxidative stress after hypoxia exposure is not limited to croaker brains because PC content, an indirect measure of ROS and oxidative stress [19], was significantly elevated in the livers of croaker after both environmental and laboratory hypoxia exposure. The observation that the PC content in croaker livers was increased within 1 day of laboratory exposure to hypoxia conditions is in agreement with the findings in grouper after the same period of hypoxia exposure [37]. This increase in hepatic PC content in grouper was accompanied by a significant increase in hepatic *igfbp-1* mRNA expression in *in vitro* [37]. Recently, we have shown that long-term hypoxia exposure increases superoxide radical (O_2_^•-^, an index of ROS) production which is accompanied by a significant increase in *hif-α*s, *nNOS* and *igfbp-1* mRNA and protein levels in croaker brain and liver tissues *in vivo*, respectively [15, 16]. Based on the current results and our previous studies [14–16], a model is proposed of the potential pathways by which hypoxia could potentially upregulate gene expression and biochemical biomarkers in croaker (Fig 7C). In the model, hypoxia-induced upregulation of croaker *hif-α*s, *nNOS*, *igfbp* transcripts and ROS and reactive nitrogen species (RNS) may occur through multiple pathways and intermediaries.

## Conclusion

The current results provide the first comprehensive evidence, to our knowledge, that *hif-α*s, *nNOS and ifgbp-1* transcripts and PC contents may be useful biomarkers of chronic environmental hypoxia exposure in a marine teleost species. In addition to indicating exposure to hypoxia in fish, these molecular and biochemical responses also suggest some of the likely adaptive and pathological impacts of environmental hypoxia exposure, such as marked changes in gene regulation, including modulation of glycolytic metabolic pathway, growth suppression, and oxidative damage and impaired functions in neural and hepatic tissues [40–42]. Thus, measurement of these biomarkers can also indicate potential adverse effects of environmental hypoxia exposure in fish populations.

## Supporting information

S1 Table Physio-chemical parameters at the station sampled Atlantic croaker in the northern Gulf of Mexico in August 2007, July 2008 and July-August 2012. (PDF)

S2 Table Primer sequences used in quantitative real-time PCR for mRNA expression analyses. (PDF)

S3 Table Physio-chemical parameters at the station sampled in the northern Gulf of Mexico by NOAA from July to September, 2007. (PDF)

S4 Table Physio-chemical parameters at the station sampled in the northern Gulf of Mexico by EPA in May 04–06 and August 20, 2007. (PDF)

S5 Table Physio-chemical parameters at the station sampled in the northern Gulf of Mexico in June 12–15, 2012. (PDF)

S6 Table Physio-chemical parameters at the station sampled in the northern Gulf of Mexico in July and August, 2012. (PDF)

S1 Fig Expression of *hif-1β and hif-2β* mRNA in Atlantic croaker exposed to environmental hypoxia. (PDF)

S2 Fig Expression of *hif-1β and hif-2β* mRNA in Atlantic croaker exposed to laboratory hypoxia. (PDF)

S3 Fig Expression of *igfbp-2* mRNA in Atlantic croaker exposed to environmental hypoxia. (PDF)

S4 Fig Hepatosomatic index in Atlantic croaker collected environmental hypoxia. (PDF)

S5 Fig Expression of *hif-1α* mRNA levels in croaker brains exposed to laboratory hypoxia. (PDF)
