# Supplementary material for: Molecular and biochemical responses of hypoxia exposure in Atlantic croaker collected from hypoxic regions in the northern Gulf of Mexico
Source: PLoS One. 2017 Sep 8;12(9):e0184341. doi: 10.1371/journal.pone.0184341 (PMC5590906; doi:10.1371/journal.pone.0184341)
Supplement: S1 Table — (PDF) [file pone.0184341.s001.pdf]

S1 Table. Physio-chemical parameters at the station sampled Atlantic croaker in the northern Gulf of Mexico in August 2007, July 2008 and July-August 2012.

Sampling station: N1 (29º26.484’N, 88º44.141’W)

Date	Bottom DO (mg/L)	Bottom salinity (ppm)	Depth (m)	Temperature (ºC)
August 13, 2007	4.2	36.4	23.0	23.8
July 14, 2008	3.6	36.4	22.1	20.7
August 03, 2013	4.6	36.3	23.6	22.2

Sampling station: N2 (29º31.508’N, 88º39.173’W)

Date	Bottom DO (mg/L)	Bottom salinity (ppm)	Depth (m)	Temperature (ºC)
August 13, 2007	3.8	36.4	22.5	23.9
July 14, 2008	4.8	36.5	21.9	20.9
August 03, 2012	4.9	36.3	23.4	22.4

Sampling station: C8 (28º45.308’N, 90º18.514’W)

Date	Bottom DO (mg/L)	Bottom salinity (ppm)	Depth (m)	Temperature (ºC)
August 10, 2007	1.2	36.1	21.4	21.7
July 13, 2008	0.8	36.4	26.6	22.3
July 31, 2012	3.2	36.1	22.0	25.6

Sampling station: C9 (28º45.883’N, 90º13.265’W)

Date	Bottom DO (mg/L)	Bottom salinity (ppm)	Depth (m)	Temperature (ºC)
August 13, 2007	1.8	36.4	20.6	24.1
July 13, 2008	2.6	36.4	31.5	21.7
July 31, 2012	3.8	36.2	28.0	25.1

Sampling station: F3 (28º52.997’N, 91º37.094’W)

Date	Bottom DO (mg/L)	Bottom salinity (ppm)	Depth (m)	Temperature (ºC)
August 11, 2007	1.0	36.0	19.0	26.0
August 04, 2012	4.6	35.8	17.2	27.8

Sampling station: F4 (28º47.057’N, 91º37.026’W)

Date	Bottom DO (mg/L)	Bottom salinity (ppm)	Depth (m)	Temperature (oC)
August 11, 2007	2.4	36.0	24.0	26.0
August 04, 2012	4.5	36.0	22.0	27.3
