# Supplementary material for: Molecular and biochemical responses of hypoxia exposure in Atlantic croaker collected from hypoxic regions in the northern Gulf of Mexico
Source: PLoS One. 2017 Sep 8;12(9):e0184341. doi: 10.1371/journal.pone.0184341 (PMC5590906; doi:10.1371/journal.pone.0184341)
Supplement: S2 Table — (PDF) [file pone.0184341.s002.pdf]

S2 Table. Primer sequences used in quantitative real-time PCR for mRNA expression analyses.

Candidate gene	Accession no.	Sense primer (5'- 3')	Anti-sense primer (5'- 3')
[1]*hypoxia inducible factor-1α*	DQ363931	AGACCGAGGATGTGAAACCA	GCCCGAGTGAACAGTTTGAT
[1]*hypoxia inducible factor-2α*	DQ363932	ATCGCATGCTGGCTAAGAAC	CGAAGTCGAGGGAAATGATG
[2]*insulin-like growth factor binding protein-1*	HQ738525	ACACCTGAAGAATGCCAACC	TGATGTTGGGCAGTGACAGT
[2]*insulin-like growth factor binding protein-2*	HQ738526	GGGATCAAAAGCAGGATGAA	GGGGTTAACACACCAGCACT
hypoxia inducible factor-1ß	DQ376248	TCCACCCAGATGACACAGAA	GGGGCCAGGATTTGATGTAT
hypoxia inducible factor-2ß	DQ376249	GATACAGGAATGGCCTTGGA	CTGGTATCCGATGACGTTGA
[3]*nitric oxide synthase*	KM067455	CTGGAGACTGGGTGTGGATT	TCTTGAATCCGATCGCTCTT

[1]Rahman MS, Thomas P. 2007 Molecular cloning, characterization and expression of two hypoxia-inducible transcription factors (HIF-1α and HIF-2α) in a hypoxia tolerant marine teleost, Atlantic croaker (*Micropogonias undulatus*). *Gene* **396**, 273-282. (doi:10.1016/j.gene.2007.03.009)

[2]Rahman MS, Thomas P. 2011. Characterization of three IGFBPs in Atlantic croaker and their regulation during hypoxic stress: potential mechanisms of their upregulation by hypoxia. *Am J Physiol Endocrinol Metab* 301, E637-E648. (doi: 10.1152/ajpendo.00168.2011)

[3]Rahman MS, Thomas P. 2015. Molecular characterization and hypoxia-induced upregulation of neuronal nitric oxide synthase in Atlantic croaker: reversal by antioxidant and estrogen treatments. *Comp Biochem Physiol Part A: Mol Integ Physiol* 185:91-106. (doi:10.1016.j.cbpa.2015.03.013)
