# Supplementary material for: Molecular and biochemical responses of hypoxia exposure in Atlantic croaker collected from hypoxic regions in the northern Gulf of Mexico
Source: PLoS One. 2017 Sep 8;12(9):e0184341. doi: 10.1371/journal.pone.0184341 (PMC5590906; doi:10.1371/journal.pone.0184341)
Supplement: S3 Table — (PDF) [file pone.0184341.s003.pdf]

S3 Table. Physio-chemical parameters at the station sampled in the northern Gulf of Mexico by NOAA from July to September, 2007*.

Sampling station: C5			
Date	July 23	August 15	September 12
Bottom DO (mg/L)	0.3	0.7	0.4
Bottom salinity (ppm)	35.4	35.5	35.4
Depth (m)	15.9	16.3	16.2
Temperature (°C)	27.7	28.5	29.0
Sampling station: C6			
Date	July 23	August 15	September 12
Bottom DO (mg/L)	0.3	1.4	0.6
Bottom salinity (ppm)	35.6	35.6	35.8
Depth (m)	18.9	18.8	19.5
Temperature (°C)	27.9	28.3	28.5
Sampling station: C7			
Date	July 23	August 15	September 12
Bottom DO (mg/L)	0.5	0 .4	0.4
Bottom salinity (ppm)	35.9	35.7	35.9
Depth (m)	20.5	20.3	20.5
Temperature (°C)	27.5	27.6	27.7
Sampling station: F3			
Date	July 25		September 11
Bottom DO (mg/L)	0.5		6.1
Bottom salinity (ppm)	35.7		32.7
Depth (m)	19.2		19.4
Tamprature (°C)	27.8		30.4
Sampling station: F4			
Date	July 25		September 11
Bottom DO (mg/L)	3.1		2.3
Bottom salinity (ppm)	35.9		35.4
Depth (m)	24.4		23.7
Temperature (°C)	27.9		29.3
Sampling station: F5			
Date	July 25		September 11
Bottom DO (mg/L)	2.4		4.5
Bottom salinity (ppm)	36.1		36.2
Depth (m)	29.7		29.4
Temperature (°C)	26.6		28.8

*Physio-chemical parameters were generously provided by Dr. Andy Rose Parsons, Chief Scientist, NOAA/NESDIS/NODC /NCDDC, Stennis Space Center, MS, 39529, USA. The physio-chemical parameters are also available in the website: http://www.nodc.noaa.gov/archive/arc0025/0060060/01-version/data/1-data/H07YSI6820.txt website.
