# Supplementary material for: Molecular and biochemical responses of hypoxia exposure in Atlantic croaker collected from hypoxic regions in the northern Gulf of Mexico
Source: PLoS One. 2017 Sep 8;12(9):e0184341. doi: 10.1371/journal.pone.0184341 (PMC5590906; doi:10.1371/journal.pone.0184341)
Supplement: S4 Table — (PDF) [file pone.0184341.s004.pdf]

S4 Table. Physio-chemical parameters at the station sampled in the northern Gulf of Mexico by EPA in May 04-06 and August 20, 2007*.

Sampling station	Depth (m)	Temperature (oC)	Salinity (ppm)	Dissolved oxygen (mg/L)
Date: May 04-06, 2007				
C5	15.0	22.5	35.6	0.38
C6	19.0	22.3	35.7	1.83
C7	20.0	22.1	35.7	2.09
F3	18.0	21.8	35.2	1.67
F4	23.0	21.3	35.9	3.14
F5	28.0	21.3	36.3	4.84
Date: August 20, 2007				
Sampling station	Depth (m)	Temperature (oC)	Salinity (ppm)	Dissolved oxygen (mg/L)
C5	14.0	28.7	35.5	0.11
C6	19.0	28.2	35.8	0.15
C7	19.0	27.9	35.9	0.14
F3	19.0	28.5	35.5	0.09
F5	29.0	25.6	36.3	0.13

*Physio-chemical parameters were generously provided by Dr. Michael C. Murrell, Research Ecologist, US EPA Gulf Ecology Division, 1 Sabine Island Drive, Gulf Breeze, FL 32561, USA.
