# Supplementary material for: Molecular and biochemical responses of hypoxia exposure in Atlantic croaker collected from hypoxic regions in the northern Gulf of Mexico
Source: PLoS One. 2017 Sep 8;12(9):e0184341. doi: 10.1371/journal.pone.0184341 (PMC5590906; doi:10.1371/journal.pone.0184341)
Supplement: S5 Table — (PDF) [file pone.0184341.s005.pdf]

S5 Table. Physio-chemical parameters at the station sampled in the northern Gulf of Mexico in June 12-15, 2012*.

Sampling station	Depth (m)	Temperature (°C)	Salinity (ppm)	Dissolved oxygen (mg l^{-1})
A02 (28.973875 lat, -89.945235 long)	28.62	23.99	37.16	3.2
A12 (29.03321 lat, -94.05000833 long)	18.42	27.47	33.59	5.61
Sampling station	Depth (m)	Temperature (oC)	Salinity (ppm)	Dissolved oxygen (mg l^{-1})
L042 (28.69.650 lat, -94.29604333 long)	33.91	23.55	36.45	3.73
L062 (29.013575 lat, -93.39918667 long)	22.56	25.88	35.12	3.47
L132 (28.556608 lat, -90.465575 long)	30.0	21.82	36.42	5.01
L162 (28.9189517 lat, -89.5104133 long)	43.59	22.45	36.43	4.94

*Physio-chemical parameters were generously provided by Dr. G. Chistopher Shank, Lecturer, University of Texas Marine Science Institute, Texas 78373, USA.
