# Supplementary material for: Molecular and biochemical responses of hypoxia exposure in Atlantic croaker collected from hypoxic regions in the northern Gulf of Mexico
Source: PLoS One. 2017 Sep 8;12(9):e0184341. doi: 10.1371/journal.pone.0184341 (PMC5590906; doi:10.1371/journal.pone.0184341)
Supplement: S6 Table — (PDF) [file pone.0184341.s006.pdf]

S6 Table. Physio-chemical parameters at the station sampled in the northern Gulf of Mexico in July and August, 2012*.

Sampling station	Depth (m)	Temperature (oC)	Salinity (ppm)	Dissolved oxygen (mg l^{-1})
Date: July 31, 2012				
C8 (28.7703333 lat, -90.3101667 long)	21.49	25.67	36.14	3.06
C9 (28.7638333 lat, -90.22016667 long)	28.02	25.19	36.19	3.63
Date: August 02, 2012				
N1 (29.4511667 lat, -88.73216667 long)	24.79	21.94	36.4	5.16
N2 (29.5048333 lat, -88.88.6425 long)	30.0	21.82	36.42	5.01
Date: August 04, 2012				
F4 (28.7828333 lat, -91.6153333 long)	22.06	27.29	36..02	4.49

*Physio-chemical parameters were generously provided by Dr. John Mohan, Postdoctoral Research Scientist, Texas A&M University- Galveston, 200 Seawolf, Parkway, Texas 77553, USA.
