# Supplementary material for: Molecular and biochemical responses of hypoxia exposure in Atlantic croaker collected from hypoxic regions in the northern Gulf of Mexico
Source: PLoS One. 2017 Sep 8;12(9):e0184341. doi: 10.1371/journal.pone.0184341 (PMC5590906; doi:10.1371/journal.pone.0184341)
Supplement: S1 Fig — (PDF) [file pone.0184341.s007.pdf]

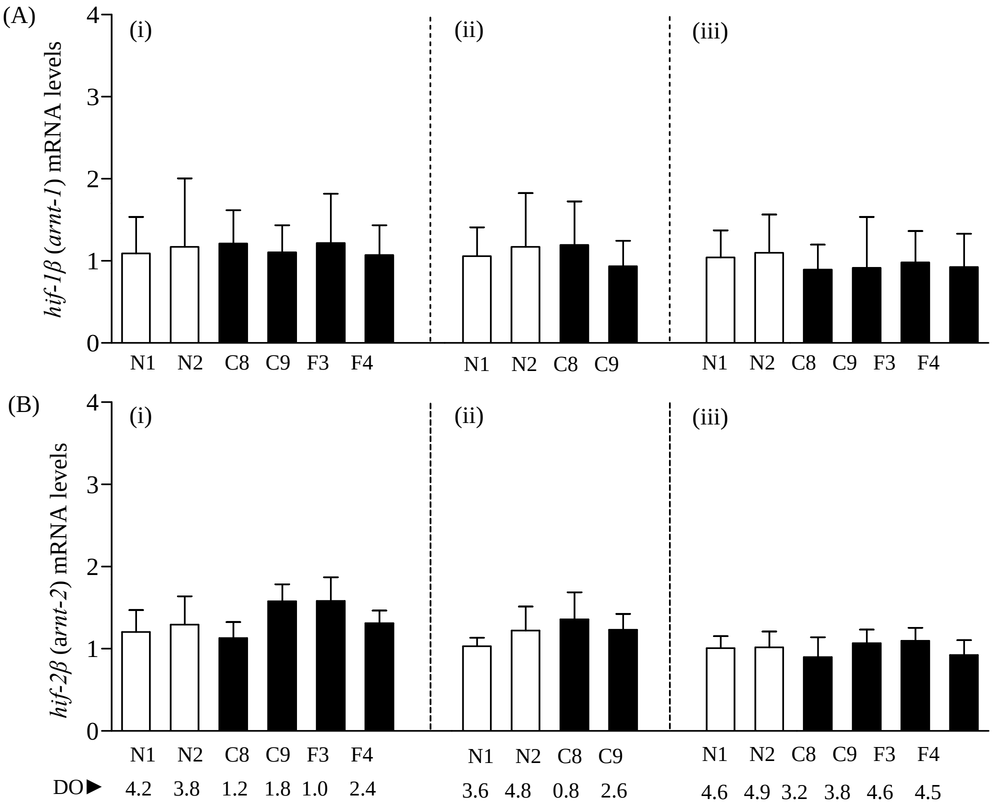

S1 Fig. Expression of *hif-1β and hif-2β* mRNA in Atlantic croaker exposed to environmental hypoxia. Relative *hif-1β* (*aryl hydrocarbon receptor nuclear translocator-1, arnt-1*) (A) and *hif-2β* (*arnt-2*) (B) mRNA levels in croaker brains collected from normoxic (N1, N2) and hypoxic (F3, F4, C8, C9) sites in August, 2007 (i); July, 2008 (ii); and August, 2012 (iii) in the northern Gulf of Mexico. The thick vertical lines represent mean values, N=8. DO, dissolved oxygen (mg/L).
