# Supplementary material for: Molecular and biochemical responses of hypoxia exposure in Atlantic croaker collected from hypoxic regions in the northern Gulf of Mexico
Source: PLoS One. 2017 Sep 8;12(9):e0184341. doi: 10.1371/journal.pone.0184341 (PMC5590906; doi:10.1371/journal.pone.0184341)
Supplement: S2 Fig — (PDF) [file pone.0184341.s008.pdf]

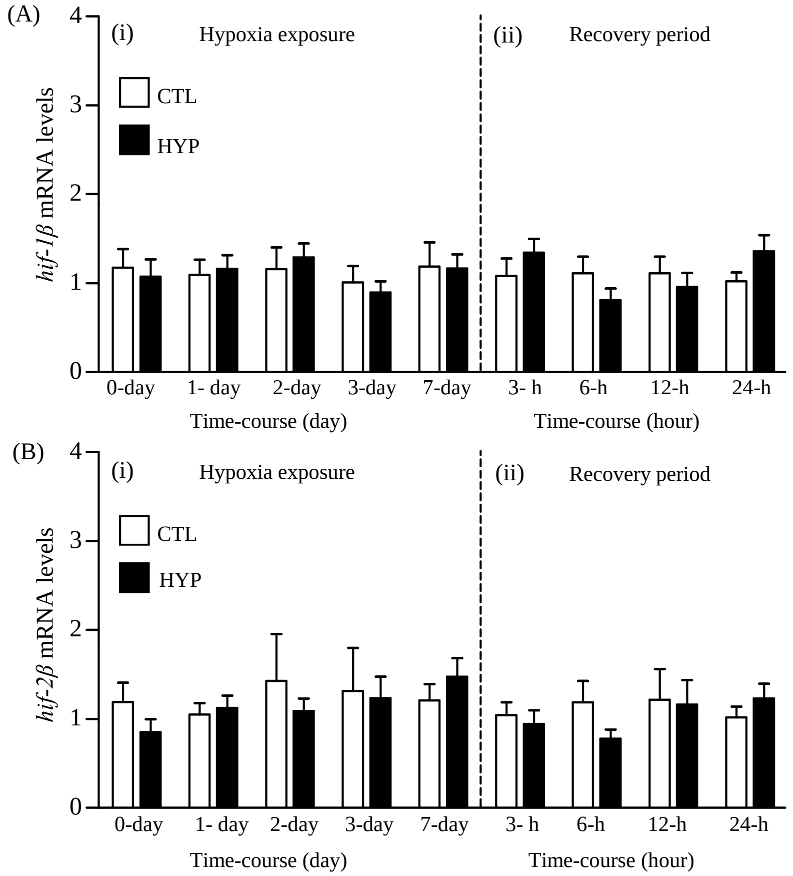


**S2 Fig. Expression of *hif-1β and hif-2β* mRNA in Atlantic croaker exposed to laboratory hypoxia.** Effects of 7-day laboratory exposure to normoxia (dissolved oxygen, DO: greater than 6 mg/L, white bars), hypoxia (HYP, DO: 1.7 mg/L, black bars) and recovery period on relative *hif-1β* (A) and *hif-2β* (B) mRNA levels in croaker brains. Each value represents the mean±SEM (N=7-8).
