# Supplementary material for: Molecular and biochemical responses of hypoxia exposure in Atlantic croaker collected from hypoxic regions in the northern Gulf of Mexico
Source: PLoS One. 2017 Sep 8;12(9):e0184341. doi: 10.1371/journal.pone.0184341 (PMC5590906; doi:10.1371/journal.pone.0184341)
Supplement: S3 Fig — (PDF) [file pone.0184341.s009.pdf]

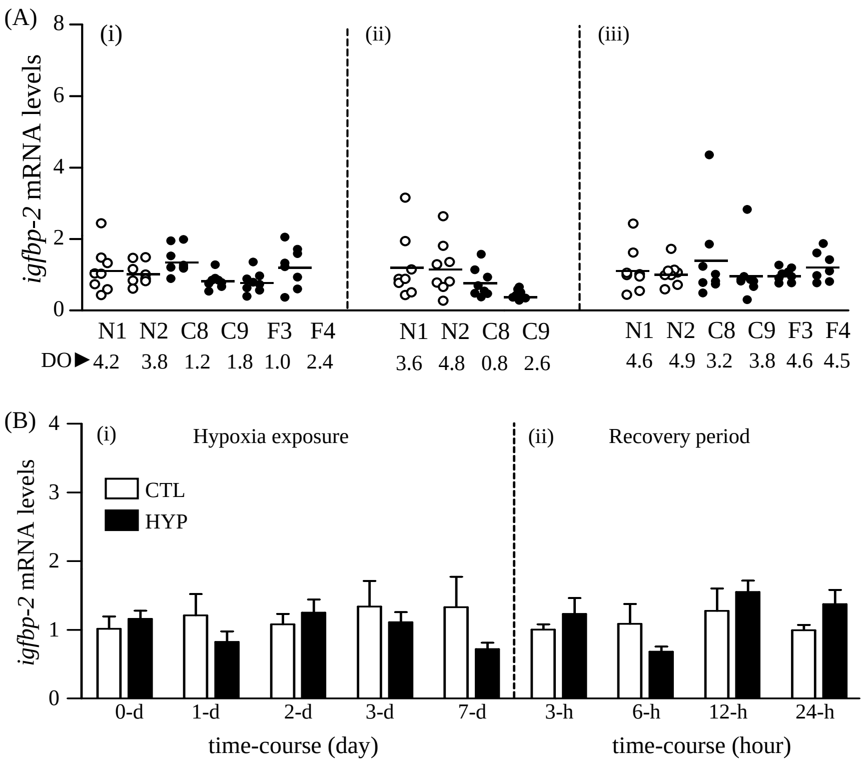

S3 Fig. Expression of *igfbp-2* mRNA in Atlantic croaker exposed to environmental hypoxia. (A) *igfbp-2* mRNA levels in croaker livers collected from normoxic (N1, N2) and hypoxic (F3, F4, C8, C9) sites in August, 2007 (*i*); July, 2008 (*ii*); and August, 2012 (*iii*) in the northern Gulf of Mexico. DO, dissolved oxygen (mg/L). The thick vertical lines represent mean±SEM (N= 7-8). (B) Expression of *igfbp-2* mRNA levels in croaker livers exposed to laboratory hypoxia. Effects of 7-day laboratory exposure to normoxia (dissolved oxygen, DO: <6 mg/L, white bars), hypoxia (HYP, DO: 1.7 mg/L, black bars) and recovery period on *igfbp-2* mRNA levels in croaker livers. Each value represents the mean±SEM (N=7-11).
