# Supplementary material for: Molecular and biochemical responses of hypoxia exposure in Atlantic croaker collected from hypoxic regions in the northern Gulf of Mexico
Source: PLoS One. 2017 Sep 8;12(9):e0184341. doi: 10.1371/journal.pone.0184341 (PMC5590906; doi:10.1371/journal.pone.0184341)
Supplement: S4 Fig — (PDF) [file pone.0184341.s010.pdf]

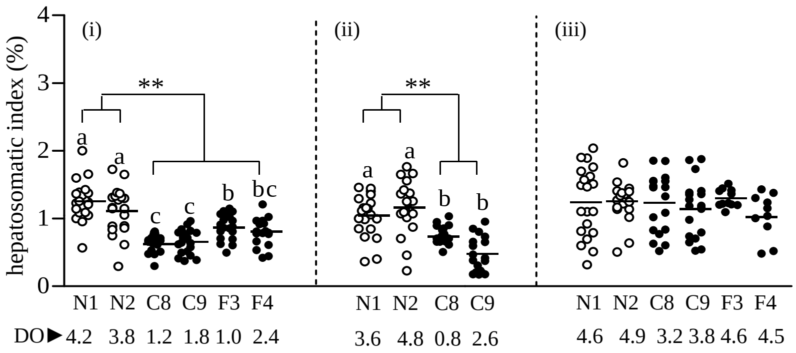

S4 Fig. Hepatosomatic index in Atlantic croaker collected environmental hypoxia. Croaker collected from normoxic (N1, N2) and hypoxic (F3, F4, C8, C9) sites in August, 2007 (i); July, 2008 (ii); and August, 2012 (iii) in the northern Gulf of Mexico. DO, dissolved oxygen (mg/L). The thick vertical lines represent mean±SEM (N= 10-20). Asterisks denote significant differences between normoxic (reference) and hypoxic sites (nested ANOVA, **p<0.01). Individual site differences are indicated with different letters (Fisher's PLSD, p<0.05).
