# Supplementary material for: Molecular and biochemical responses of hypoxia exposure in Atlantic croaker collected from hypoxic regions in the northern Gulf of Mexico
Source: PLoS One. 2017 Sep 8;12(9):e0184341. doi: 10.1371/journal.pone.0184341 (PMC5590906; doi:10.1371/journal.pone.0184341)
Supplement: S5 Fig — (PDF) [file pone.0184341.s011.pdf]

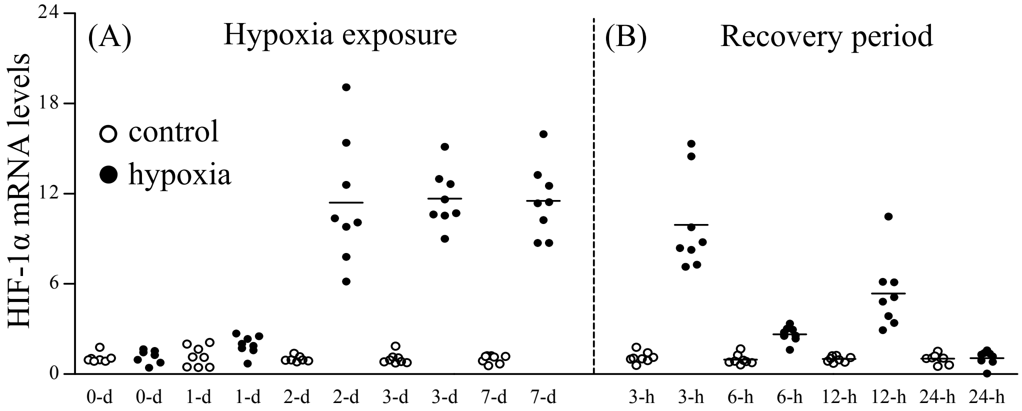

S5 Fig. Expression of *hif-1α* mRNA levels in croaker brains exposed to laboratory hypoxia. Effects of 7-day laboratory exposure to normoxia control (dissolved oxygen, DO: <6 mg l^{-1}, white circles), hypoxia (HYP, DO: 1.7 mg l^{-1}, black circles) (A) and recovery period on relative *hif-1α* (B) mRNA levels in croaker brain. The thick vertical lines represent mean values, N=7-8.
